# The Role of Adaptor Proteins in the Biology of Natural Killer T (NKT) Cells

**DOI:** 10.3389/fimmu.2019.01449

**Published:** 2019-06-25

**Authors:** Evelyn Gerth, Jochen Mattner

**Affiliations:** Mikrobiologisches Institut - Klinische Mikrobiologie, Immunologie und Hygiene, Universitätsklinikum Erlangen, Friedrich-Alexander Universität (FAU) Erlangen-Nürnberg, Erlangen, Germany

**Keywords:** NKT cells, CD1d, adaptor proteins, T cell receptor, NK cell receptor, differentiation, polarization

## Abstract

Adaptor proteins contribute to the selection, differentiation and activation of natural killer T (NKT) cells, an innate(-like) lymphocyte population endowed with powerful immunomodulatory properties. Distinct from conventional T lymphocytes NKT cells preferentially home to the liver, undergo a thymic maturation and differentiation process and recognize glycolipid antigens presented by the MHC class I-like molecule CD1d on antigen presenting cells. NKT cells express a semi-invariant T cell receptor (TCR), which combines the Vα14-Jα18 chain with a Vβ2, Vβ7, or Vβ8 chain in mice and the Vα24 chain with the Vβ11 chain in humans. The avidity of interactions between their TCR, the presented glycolipid antigen and CD1d govern the selection and differentiation of NKT cells. Compared to TCR ligation on conventional T cells engagement of the NKT cell TCR delivers substantially stronger signals, which trigger the unique NKT cell developmental program. Furthermore, NKT cells express a panoply of primarily inhibitory NK cell receptors (NKRs) that control their self-reactivity and avoid autoimmune activation. Adaptor proteins influence NKT cell biology through the integration of TCR, NKR and/or SLAM (signaling lymphocyte-activation molecule) receptor signals or the variation of CD1d-restricted antigen presentation. TCR and NKR ligation engage the SH2 domain-containing leukocyte protein of 76kDa slp-76 whereas the SLAM associated protein SAP serves as adaptor for the SLAM receptor family. Indeed, the selection and differentiation of NKT cells selectively requires co-stimulation via SLAM receptors. Furthermore, SAP deficiency causes X-linked lymphoproliferative disease with multiple immune defects including a lack of circulating NKT cells. While a deletion of slp-76 leads to a complete loss of all peripheral T cell populations, mutations in the SH2 domain of slp-76 selectively affect NKT cell biology. Furthermore, adaptor proteins influence the expression and trafficking of CD1d in antigen presenting cells and subsequently selection and activation of NKT cells. Adaptor protein complex 3 (AP-3), for example, is required for the efficient presentation of glycolipid antigens which require internalization and processing. Thus, our review will focus on the complex contribution of adaptor proteins to the delivery of TCR, NKR and SLAM receptor signals in the unique biology of NKT cells and CD1d-restricted antigen presentation.

## Introduction

Specific and appropriate intercellular interactions or the communication of cells with their environment requires the integration and coordination of multiple signaling pathways. Adaptor proteins contain a series of protein-binding sites that link respective interaction partners to each other and facilitate the generation of larger signaling complexes (1). This is, for example, pivotal for the delivery of signals from the T cell receptor (TCR) which plays a critical role in T cell biology (2).

There exist several T cell populations with distinct functions (3). Alpha beta (αβ) T cells, for example, termed conventional (αβ) T cells, are predominantly part of the adaptive immune system and display a large TCR diversity. TCR ligation by self-peptides embedded in major histocompatibility complex (MHC) molecules on antigen-presenting cells (APCs) in the thymus determines the fate of developing conventional T cells. Weak TCR signals perpetuate positive selection whereas strong, agonist, signals support the removal of potentially self-reactive TCRs through negative selection (4). The resulting diverse TCR repertoire endows conventional T cells to respond to foreign antigens in the periphery upon exit from the thymus. NKT cells can be divided into two distinct subpopulations.

In contrast, mucosa-associated semi-invariant T (MAIT) cells, gamma delta (γδ) T cells and natural killer T (NKT) cells express semi-invariant TCRs with limited diversity and react rapidly to conserved self and/or microbial ligands. Most of these cells acquire memory cell features during thymic maturation and exhibit unique patterns of migration into peripheral, frequently non-lymphoid tissues where they become resident, regulate tissue homeostasis and/or fight infection (5). These innate(-like) T lymphocytes display also several other innate-like characteristics and are therefore considered to be mainly part of the innate immune system. Distinct from conventional T cells, innate(-like) lymphocytes recognize higher affinity and avidity antigens through their TCR, which has been suggested to deliver substantially stronger signals (4, 6). Thus, the TCR signal threshold for negative selection is higher. However, it is not completely understood how unconventional T cell precursors escape negative selection despite agonist signaling. Thus, adaptor proteins might play a pivotal role in the tight control of TCR signals as they tie multiple and complex intracellular pathways. Indeed, some adaptor proteins are specifically important for innate(-like) lymphocytes, and a lack of specific adaptor proteins impairs or even selectively inhibits the selection of these frequently autoreactive cell subsets. In detail, we will discuss here the impact of adaptor proteins on the biology of natural killer T (NKT) cells. We will focus thereby on type 1 or invariant NKT cells, which we will refer to as iNKT cells hereinafter.

## Natural Killer T (NKT) Cells and CD1d-Mediated Antigen Presentation

Natural killer T (NKT) cells belong to the group of innate(-like) unconventional T cells. They explosively release various cytokines and chemokines upon TCR engagement and thus, exhibit powerful immunomodulatory properties. NKT cells can be divided into two distinct lineages, namely type 1 or invariant NKT cells and type 2 NKT cells. Type 2 NKT cells exhibit a more diverse TCR repertoire. In contrast, type 1 or invariant NKT cells—hereinafter referred to as iNKT cells—express a semi-invariant canonical T cell receptor (TCR), which combines the Vα14-Jα18 chain with the Vbeta2, Vbeta7, or Vbeta8 chain in mice and the Vα24-Jα18 chain with the Vβ11 chain in humans. Simultaneously, they carry a wide range of activating and inhibitory NK cell receptors (NKRs) on their surface (7). The inhibitory NKRs presumably control the self-reactivity of iNKT cells and avoid autoimmune activation (8, 9). Vice versa, the NKT cell TCR shapes the pattern of NKR expression, as exemplified for Ly49 receptors (10). Furthermore, balanced signaling through activating and inhibitory NKRs might influence the developmental program of iNKT cells (11). As NKR signaling engages also adaptor proteins, the propagation of signal transduction through adaptor molecules is in particular critical for diverse ranges of cellular processes in iNKT cells.

**Figure 1:**
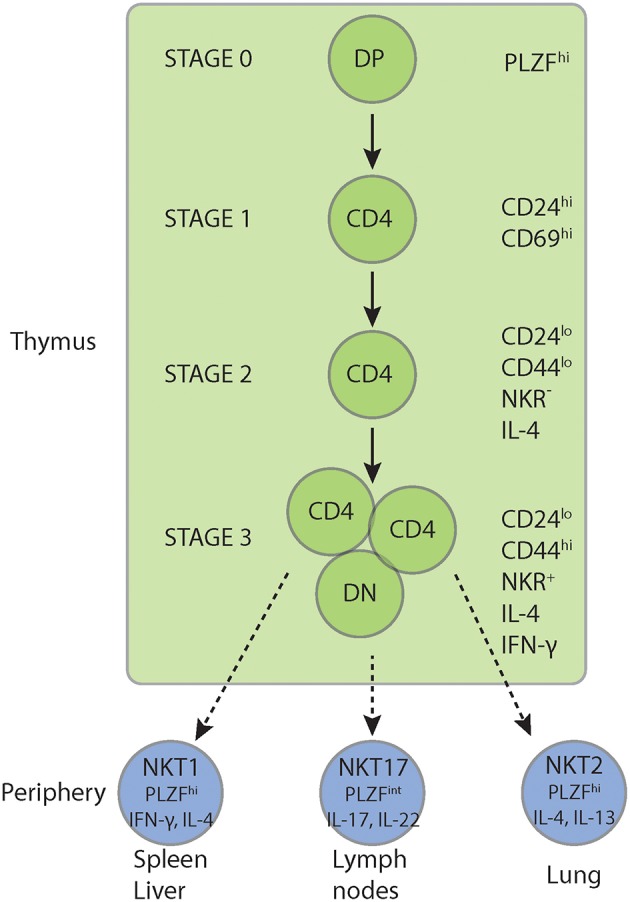
iNKT cell development. iNKT cells express PLZF upon positive selection and undergo a unique intrathymic maturation, expansion, and differentiation program. This includes multiple rounds of intrathymic cell divisions, the acquisition of a memory phenotype, the activation of Th1, Th2 and Th17 cytokine genes and the expression of a panoply of NKRs. Furthermore, iNKT cells branch into three polarized subsets, NKT1, NKT2, and NKT17 cells before egress into the periphery. In the periphery NKT1 cells home preferentially to the spleen and liver while NKT2 and NKT17 cells mainly populate the lung and peripheral lymph nodes, respectively. Although TCR signal strength has been implicated in the polarization of the three iNKT cell sublineages and the regulation of PLZF expression, the intrathymic branching traits and cellular and molecular mechanisms of sublineage diversification are still under investigation. NKR, NK lineage receptors including NK1.1, Ly49, NKG2D, CD94, DX5; PLZF, promyelocytic leukemia zinc finger.

In contrast to conventional T cells, iNKT cells respond to glycolipid antigens and home predominantly to the liver (12). Unlike the development of conventional T cell, the selection of iNKT cells requires antigen presentation by double-positive thymocytes rather than thymic epithelial cells (13–17). iNKT cells are selected on high-affinity self-glycolipid ligands presented by the MHC class I-like molecule CD1d (18) which triggers their unique developmental program (19). Their selection uniquely requires co-stimulation via SLAM (signaling lymphocyte-activation molecule) family members and the tyrosine kinase Fyn (20–32) as discussed below. Once selected in stage 0, iNKT cells pass through complex activation, expansion, maturation and differentiation processes, termed stages 1–3 (Figure 1). These include the induction and regulation of promyelocytic leukemia zinc finger PLZF, the iNKT cell lineage transcription factor, multiple rounds of intrathymic cell divisions, the acquisition of a memory phenotype, the activation of cytokine gene loci, and the expression of multiple NKRs over the course of several weeks (7, 33). Although associated with their development (33, 34), PLZF is not unique to iNKT cells and also expressed in innate lymphoid cells (ILCs), mucosa-associated semi-invariant T (MAIT) cells and subsets of γδ T cells (35–37).

Furthermore, iNKT cells differentiate into three polarized subsets, NKT1, NKT2, and NKT17 cells (38) before egress into the periphery (Figure 1). Although TCR signal strength has been implicated in the polarization of the three iNKT cell sublineages and the regulation of PLZF expression (39), the intrathymic branching traits and cellular and molecular mechanisms of sublineage diversification are still under investigation. TCR-specific signals contribute also to the tissue distribution and phenotypic presentation of iNKT cells (40, 41). Although the signal delivered through the iNKT cell TCR is stronger than for the conventional T cell TCR (6, 42–44), the role of the TCR signal strength in iNKT cell lineage commitment and differentiation is still under investigation.

Next to α/β-TCR^+^ iNKT cells CD1d-restricted γ/δ T cells also respond to (glycol-)lipid antigens (45). These γ/δ NKT cells express γ1.1 and δ6.3 chains and the promyelocytic leukemia zinc finger *(*PLZF*)*, the lineage transcription factor of NKT cells. Further comparisons of γ/δ- with α/β-TCR expressing NKT cells revealed also converging patterns of cytokine, gene and cell surface marker expression implying similar differentiation programs in both NKT cell subsets (33, 34, 37, 46–48). Thus, several observations obtained with α/β-TCR^+^ iNKT cells, might be reflected in the biology of CD1d-restricted γ/δ T cells.

Another feature of iNKT cells distinct from conventional T cells is the recognition of glycolipid antigens presented by CD1d. CD1d molecules are assembled in the endoplasmatic reticulum (ER) as non-covalently linked heterodimers of an isotype-specific heavy chain and β-2-microglobulin (β2m). During its assembly in the ER, CD1d incorporates endogenous lipids and traffics to the plasma membrane. While certain lipids can load onto CD1d directly at the cell surface, CD1d with its hydrophobic binding groove of intermediate size usually has to recycle into late endosomal and lysosomal compartments for efficient antigen exchange and loading (49, 50). Upon trafficking back to the cell surface, antigens are presented by CD1d to NKT cells (51, 52).

## Adaptor Proteins in iNKT Cell Biology

Adaptor molecules are multi-domain proteins lacking intrinsic catalytic activity, functioning instead by nucleating molecular complexes during signal transduction (53). Several adaptor proteins influence iNKT cell selection, differentiation and activation, either intrinsically or indirectly through interference with CD1d-mediated antigen presentation. For example, one of the pivotal molecules engaged upon TCR ligation is the intracellular adaptor protein slp-76. While the complete absence of slp-76 (54–56) or of its N-terminal region (57) leads to a lack of all peripheral T cell populations, selective mutations in the SH2 domain of slp-76 affect in particular iNKT cells (58). Most importantly and in strict contrast to conventional T cells, the selection of iNKT cells requires co-stimulation via SLAM (signaling lymphocyte-activation molecule) family members (20–24). Thus, the SLAM-associated adaptor protein (SAP) signaling pathway is selectively required for iNKT cell development. Adaptor proteins, however, can also influence CD1d expression by antigen presenting cells (APCs) and subsequently affect iNKT cell biology in an extrinsic manner. Adaptor protein complex 3 (AP-3), for example, is required for the efficient presentation of glycolipid antigens that require internalization and processing (59).

### The slp-76 Family of Adaptor Proteins

The slp-76 family of adaptors includes the SH2 domain-containing leukocyte phosphoprotein of 76 kDa (slp-76), the B cell linker protein (BLNK), and the cytokine-dependent hematopoietic cell linker (Clnk) (53). All three proteins interact with similar but not identical signaling molecules and are critical for the integration of multitudinous signal cascades downstream of immunotyrosine-based activation motif (ITAM)-bearing receptors and integrins in various hematopoietic cell populations (60). Slp-76 is expressed in T cells, monocytes/macrophages, NK cells, mast cells and platelets (61, 62). BLNK reflects the slp-76 homolog in B cells. It shares about a 33% amino acid identity, but some of its structural domains are similar to those of slp-76 (60, 63, 64). BLNK is primarily responsible for the transmission of signals through the B cell receptor (BCR). CLNK is selectively expressed in various hematopoietic cells following cytokine stimulation (65).

#### The SH2 Domain-Containing Leukocyte Phosphoprotein of 76 kDa, Slp-76

Of these three family members primarily slp-76 is pivotal for T cell development and TCR signaling (61, 62). Due to impaired signals from the pre-TCR, double negative 3 (DN3) T cells cannot transform into the double negative 4 (DN4) stage (54, 55, 57). Consequently, slp-76^−/−^ mice lack all peripheral mature T cells (57).

**Figure 2:**
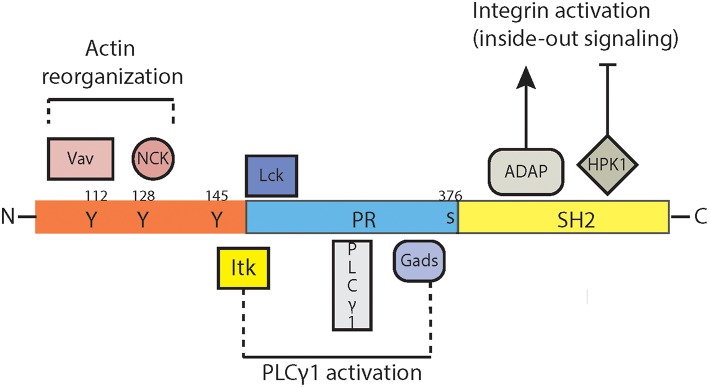
Signaling domains of slp-76. SLP76 (SRC homology 2 (SH2)-domain-containing leukocyte protein of 76 kDa) contains inducibly phosphorylated tyrosines in the amino (N) terminus, a central proline-rich (PR-) domain and a carboxy (C)-terminal SH2 domain. The N-terminal acidic domain binds to the SH2 domains of the guanine nucleotide exchange factor Vav, the adaptor protein Nck and the Tec-family kinase Itk. The subsequent signaling pathways influence predominantly the reorganization of actin. This domain of slp-76 interacts also with the phosphatidylinositol 3-kinases (PI3K) which interfere with multiple cellular functions such as proliferation, differentiation and survival. The central proline-rich domain of slp-76 interacts with the phospholipase PLCγ-1 and the adaptor molecule GADS (Grb2-related adaptor downstream of Shc). The C-terminal SH2 domain of slp-76 binds to the serine-threonine kinase HPK-1 (hematopoietic progenitor kinase 1) and to the adhesion and degranulation-promoting adaptor protein (ADAP), two molecule associated with the formation of the immunological synapse, integrin activation/expression and inside-out and outside-in signaling cascades. ADAP, adhesion- and degranulation-promoting adaptor protein; GADS, GRB2-related adaptor protein; GRB2, growth-factor-receptor-bound protein 2; HPK1, hematopoietic progenitor kinase 1; ITK, interleukin-2-inducible T-cell kinase; Lck, lymphocyte-specific protein tyrosine kinase; NCK, non-catalytic region of tyrosine kinase; PLCγ1, phospholipase Cgamma1; PI3K, phosphatidylinositol 3-kinase; PR, proline-rich; Vav, guanine nucleotide exchange factor.

The divergent functions of slp-76 are mediated by its distinct signaling domains (Figure 2). The N-terminal acidic domain contains three tyrosine residues (66) which become phosphorylated by the protein tyrosine kinase ZAP-70 upon TCR ligation (67, 68) and subsequently bind the SH2 domains of the guanine nucleotide exchange factor Vav (68–70), the adaptor protein Nck (71, 72) and the Tec-family kinase Itk (73, 74). The deletion of this N-terminal region (57) leads to a lack of all peripheral T cell populations, similar as the complete knockout of slp-76 protein (54, 55, 57). Of these three binding partners in particular Itk affects the development, maturation, cytokine production and survival of NKT cells (75–79). Itk-deficiency affected thereby not only α/β-TCR-, but also γ/δ-TCR-expressing NKT cells which in particular affect the control of Th2 responses and IgE production (80).

The central proline-rich domain of slp-76 interacts with the phospholipase PLCγ-1 (81) and the adaptor molecule GADS (Grb2-related adaptor downstream of Shc) (82). For none of these two molecules a role in NKT cell biology has been established so far.

The C-terminal SH2 domain of slp-76 binds to the serine-threonine kinase HPK-1 (hematopoietic progenitor kinase 1) (83) and to the adhesion and degranulation-promoting adaptor protein (ADAP) (84, 85). ADAP is required for thymocyte selection and TCR-mediated integrin activation (86–88). Thus, slp-76 interferes with inside-out and outside-in signaling cascades and integrin-expression (89) due to its multipoint binding with ADAP (90).

A missense mutation within the SH2-domain of slp-76 led to an accumulation of iNKT cells in the thymus and in peripheral lymph nodes. In contrast, iNKT cells were selectively reduced in the spleens and livers of mice with the same mutation, along with a reduced cytokine response, decreased levels of ADAP protein and altered integrin and NKR expression patterns (58). Although TCR signals were affected by these mutations, NKRs might contribute to the observed phenotype as this mutation affected also synapse formation and elimination of missing-self targets by natural killer (NK) cells (91). In this context, it is important to note that the tyrosine protein phosphatase SHP-1 dephosphorylates its direct substrate slp-76 (92), which reflects an important mechanism for the negative regulation of immune cell activation by inhibitory NKRs. Further studies need to delineate the mechanisms underlying the altered pattern of NKR expression in mice with this slp-76 mutation and the role of TCR signals in these processes. In addition, the specificity of this mutation for iNKT cells needs to be characterized in further detail by assessing the alterations in subsequent signaling pathways and by screening additional slp-76 mutations. Interestingly, despite exhibiting an NKR distribution that has been associated with enhanced Th1 polarization (7, 38), a simultaneous reduction of both IL-4- and IFN-γ-expression along with a reduced TCR-reactivity was observed in iNKT cells carrying this missense mutation within the SH2-domain of slp-76 (58). Thus, variations in the tissue distribution rather than the cytokine polarization are to be considered in patients with allelic mutations in TCR signaling molecules before pursuing vaccination strategies involving α-GalCer, the prototypical iNKT cell ligand as an adjuvant.

#### The Cytokine-Dependent Hematopoietic Cell Linker (clnk)

Next to cytokine driven expression clnk plays a role in Fc-epsilon R1-mediated mast cell degranulation, B cell receptor (BCR) and TCR signaling (60, 65). While not found in resting T cells, clnk is abundantly expressed in previously activated T cells (65). Similar to slp-76, clnk consists of a tyrosine- and proline-rich amino-terminal basic domain, an SH2 domain and a carboxy-terminal tail (60). While the SH2 domains of slp-76 and clnk exhibit the highest degree of homology within their SH2 domains the sequence variations outside this region suggest that clnk might not be phosphorylated by ZAP-70 and does not associate with Vav, Nck, or GADS. Clnk can rescue TCR signals in slp-76-deficient T cells (65), but clnk itself is dispensable for T cell function and differentiation (93). Clnk might contribute to the coordination of antigen-receptor signaling and cytokine stimulation. Interestingly, clnk might mediate diverse or even opposite signals by TCRs and NKRs as it promotes iNKT cell responses, but impairs NK cell function (94). Thus, clnk might function as a molecular switch, which controls diverse immune responses in different cell populations.

### Signaling Lymphocytic Activation Molecule (SLAM) and Signaling Lymphocytic Activation Molecule-Associated Protein (SAP)

The signaling lymphocytic activation molecule (SLAM) family of cell surface receptors comprises six members named 2B4 (CD244), Ly9 (CD229), CRACCSLAM (CD150), CD84, and Ly108 (95, 96) which are exclusively expressed on hematopoietic cells. They represent homophilic receptors with the exception of 2B4, which recognizes CD48. SLAM family receptors possess an extracellular segment with two or four immunoglobulin-like domains responsible for ligand recognition, a single transmembrane region and a cytoplasmic domain. This cytoplasmic domain bears one to three inhibitory or activating immunoreceptor tyrosine-based switch motifs (ITSMs) (97).

Signaling lymphocytic activation molecule (SLAM)-associated proteins (SAPs) are adaptor molecules which contain Src homology 2 (SH2) domains. SAPs are expressed in T cells, NK cells, and iNKT cells. The SAP family of adaptors includes three members most commonly known as SAP (also named SH2D1A), Ewing's sarcoma-associated transcript-2 (EAT-2; also named SH2D1B1) and EAT-2-related transducer (ERT; also named SH2D1B2) (98). Mutations in the SAP (SH2D1A) gene located on chromosome X are responsible for X-linked lymphoproliferative disease (XLP), characterized by higher susceptibility to Epstein-Barr virus (EBV) infection, B cell lymphomas, severe immune dysregulation, a nearly complete loss of iNKT cells and an impaired humoral immunity (22, 23, 99–102). The correlation of an augmented susceptibility to EBV infections with the lack of iNKT cells together with the observation that the SLAM family receptor 2B4 exhibits defect signaling function in SAP-deficiency (103–105) suggest a key role for iNKT cells and SLAM family receptors in the immune response to EBV.

**Figure 3:**
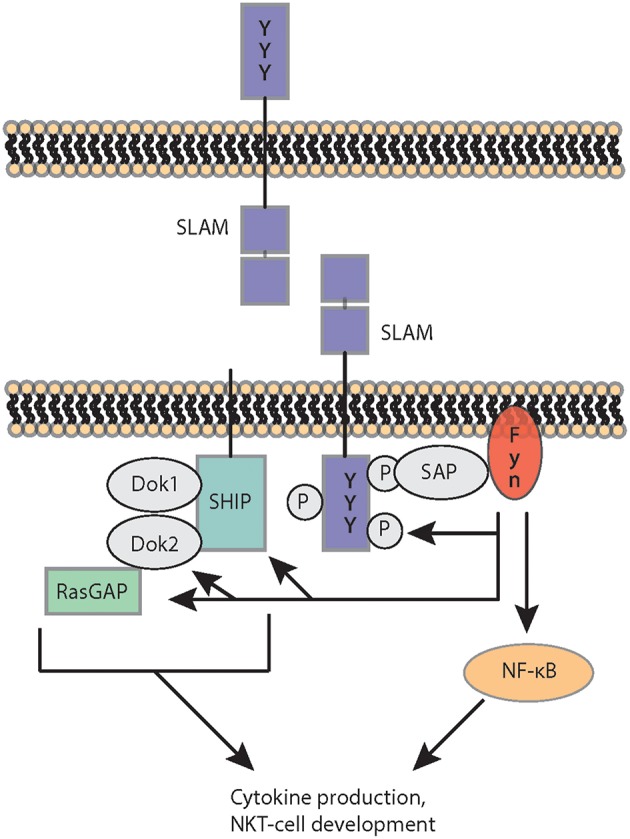
SLAM receptor signaling. SLAM receptor ligation recruits and activates via SAP the protein tyrosine kinase FYN, which phosphorylates SLAM and subsequently generates docking sites for SRC homology 2 (SH2)-domain-containing downstream adaptor proteins and enzymes. SLAM engagement also cooperates with TCR-mediated signals leading to nuclear factor-κB (NF-κB) activation, cytokine production and NKT cell development. DOK1 and 2, docking proteins 1 and 2; RasGAP, RAS–GTPase-activating protein; SAP, Signaling lymphocytic activation molecule (SLAM)-associated proteins; SLAM, signaling lymphocytic activation molecule; SHIP, SH2-domain-containing inositol−5′ phosphatase.

SAP family adaptor proteins respond through their SH2 domains to the cytoplasmic domains of SLAM family receptors by recruiting and activating the downstream tyrosine kinase Fyn (Figure 3) (106). However, SLAM family receptors can also signal through other SH2 domain–containing molecules such as the protein tyrosine phosphatases SHP-1 and SHP-2 or the SH2 domain inositol phosphatase 1 (SHIP-1), particularly in SAP deficiency (25, 97, 101, 107–111). While SAP-dependent SLAM family receptor signaling is pivotal for the selection of iNKT cells, these receptors inhibit SAP-independently follicular helper T cells and humoral immune responses (25).

iNKT cells are known to use unique signaling pathways (26). Fyn, for example, is required for iNKT cell development, but not for the differentiation of conventional T lymphocytes or NK cells (20, 21). The loss of SAP resulted in a complete absence of iNKT cells from both mice and humans. SAP-transmitted signaling events were uniquely required for the development of iNKT cells, as conventional T cells and NK cells developed normally in the absence of SAP (22, 23). The selection of iNKT cells also strictly requires co-stimulation via SLAM (signaling lymphocyte-activation molecule) family members (20–24). Homotypic interactions involving the SLAM family receptors 1 and 6 are required for iNKT cell differentiation (24). While SAP deficiency blocks positive selection at stage 0, the most immature stage of iNKT cell development (22, 23), mice lacking SLAM receptors exhibit less pronounced iNKT cell defects that appear to spare stage 0 iNKT cells (24, 25). Indeed, unlike SAP, SLAM family receptors promoted iNKT cell development and intrathymic maturation due to the restriction of TCR signal strength following positive selection and the limitation of activation induced cell death (27). This process involves the adaptor SAP-kinase Fyn complex and the protein tyrosine phosphatases SHP-1. Thus, this study uncovers important differences in SAP and SLAM signaling and highlights the complex processes underlying iNKT cell maturation and survival (112) as auto-reactive iNKT cell activation during thymic selection is thought to induce a substantially stronger TCR stimulus in comparison to that during the development of conventional T cells (6, 113). As a consequence the expression of the transcription factors Egr1 and Egr2 is strongly increased (113), which in turn directly induce PLZF, the key transcription factor controlling iNKT cell differentiation, migration, and functions (113). SAP regulates also cytokine production, expression of transcription factors, the polarization of iNKT cells favoring the development of NKT2 cells and the formation of the immunologic synapse (28, 114, 115). Furthermore, SAP expression in iNKT cells promotes cognate help to B cells (116, 117). Thus, the SLAM-associated adaptor protein (SAP) signaling pathway is selectively required for iNKT cell development and the loss of iNKT cells has been suggested to contribute to the genesis of the lethal immunodeficiency syndrome. The need for SAP-mediated signals may reflect the unique requirements for the positive selection of iNKT cells in the thymus. However, several questions remain unresolved. For example, the role of individual SLAM family receptors in cytokine polarization and iNKT cell differentiation needs to be characterized in more detail as well as the impact of subsequent signaling cascades and their interference with NKRs and TCRs. In addition, it is still unknown, whether and how TCR and SLAM family receptors interfere on a cellular and molecular level and why this is specific for iNKT cells.

### Adaptor Protein-3 (AP-3)

The hetero-tetrameric AP (adaptor protein) complexes are involved in the sorting of cargo proteins into transport vesicles that traffic between the different organelles of the cell. They are known to bind to the tyrosine or dileucine-containing sequence motifs in transmembrane proteins in order to direct their selective localization to subsets of endosomal and lysosomal compartments (118, 119). Five members, AP-1 to AP-5 and their isoforms have been characterized in this family of cytosolic complexes (118–120). In contrast to AP-4 and-5, AP-1,-2, and-3 are clathrin-associated complexes (121). AP-1 and AP-2 direct proteins from the trans-Golgi network to endosomes and recycling compartments, respectively (122, 123). AP-3 localizes membrane proteins to lysosomes, platelet-dense granules, and melanosomes (124). AP-3-deficient mice as well as Hermansky-Pudlak syndrome type 2 (HPS-2) patients with mutations in the *AP-3* gene exhibited hypopigmentation and platelet dysfunction (125–129). AP-4 mediates vesicle trafficking from the *trans*-Golgi network to endosomes or the basolateral plasma membrane. The function of AP-5 localized in late endosomes is largely unknown (121). To date, there have been no interactions between AP-1, AP4, and AP-5 with CD1d described. However, CD1d directly interacts with AP-2, which targets the endosomal compartment, and AP-3, which targets the lysosomal compartment (59, 130). Indeed, AP-2 restrains iNKT cell activation due to the regulation of CD1d internalization (131), and a connection of AP-2 with autophagy as a regulator of iNKT cell activation, development and survival is currently under investigation. In this context, a deletion of the essential autophagy gene *Atg7* abrogated thymic iNKT cell development and peripheral iNKT cell functions in a cell-intrinsic manner (132, 133). Unexpectedly, however, *Atg7*-deficient thymocytes and bone marrow-derived DCs exhibited no defect in the presentation of glycolipid antigens, implying distinct differences in the mechanisms how AP-2 and autophagy genes affect iNKT cell development and activation that need to be dissected in the future.

In contrast, numerous studies have investigated the interaction of AP-3 and CD1d. Since CD1d recycles between the cell membrane and the lysosome back and forth, AP-3 interferes with glycolipid metabolism and CD1d-mediated (glyco-)lipid antigen presentation (134). Indeed, it was shown that AP-3 is required for the efficient presentation of glycolipid antigens that require internalization and processing (59, 135). AP-3 interacts with CD1d, but does not affect MHC II presentation (59, 135–137). Cells from AP-3-deficient mice show increased cell surface expression of CD1d but decreased expression in late endosomes. Consequently, AP-3-deficient splenocytes present glycolipids to iNKT cells less efficiently. Furthermore, AP-3–deficient mice exhibit significantly reduced iNKT cell numbers. The simultaneous analysis of CD1d mutants with alterations in the cytoplasmic tail to AP-3-knockout mice proved also that CD1d molecules in lysosomes are functional in antigen presentation (59, 130). iNKT cell numbers are reduced in patients with Hermansky-Pudlak syndrome type 2 (HPS-2) (138) and iNKT cell defects have been also associated with the susceptibility to infections and lymphoma in patients with this homozygous genomic AP-3 deletion (139). Thus, in summary these studies showed that the localization of CD1d to late endosomes or lysosomes is required for both (glycol-)lipid antigen presentation and the subsequent development of iNKT cells. These reports also demonstrated that different pathways mediate the intracellular trafficking of MHC II and CD1 molecules, which both scavenge late endosomes or lysosomes.

## Conclusion

**Figure 4:**
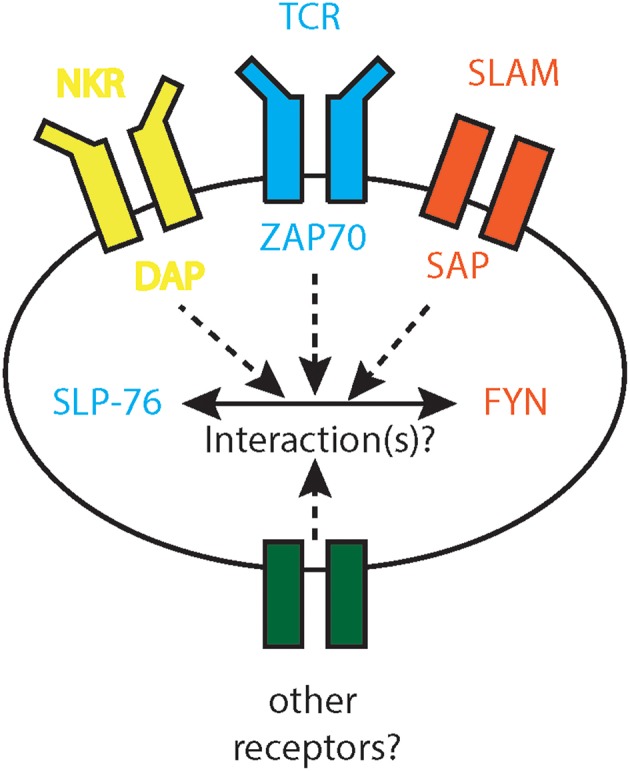
Interactions of SAP and slp-76. SLAM receptor signaling cooperates with TCR and NKR signals. While TCR and NKR signal through slp-76, SLAM receptors utilize SAP and Fyn. It is unknown to date whether slp-76 and SAP interact, whether these three receptor classes combine slp-76 and SAP signals and whether other receptors share similar signaling pathways. DAP, DNAX activation adaptor protein; Fyn, SRC family tyrosine kinase; NKR, NK cell receptor; SAP, Signaling lymphocytic activation molecule (SLAM)-associated proteins; SLAM, signaling lymphocytic activation molecule; slp-76, SRC homology 2 (SH2)-domain-containing leukocyte protein of 76 kDa; TCR, T cell receptor; ZAP-70, zeta-chain associated protein kinase 70.

Adaptor proteins play a pivotal role in the biology of CD1d-restricted iNKT cells. SAP transfers SLAM receptor signals, propagates the thymic selection of iNKT cells and induces the iNKT cell effector program (33). The SH2 domain of slp-76 influences the tissue distribution and phenotype of iNKT cells in the periphery (58). AP-3 interferes with the presentation of glycolipid antigens by CD1d (59). Thus, these three adaptor proteins engage unique functions in iNKT cells biology distinct from conventional T lymphocytes. Particularly the expression of SAP and slp-76 in iNKT cells raises the question whether these two molecules interact (Figure 4). As SLAM receptors, NKRs and TCRs share adaptor proteins for signal transmission (140, 141), it will be interesting to define the contribution of the respective receptors to the observed phenotypes. Another interesting candidate to investigate in this context is the protein tyrosine kinase SHP-1 since it also interferes with all three receptor classes (111, 116, 142–144) and localizes with slp-76 and fyn in lipid rafts (145–147), even though evidence of physical interactions of these three molecules in iNKT cells is missing. As the strength of the TCR signals influences the polarization of iNKT cell subsets (39), the role of adaptor proteins in fine-tuning intracellular signal transduction is to characterize. In addition, as SLAM receptors are pivotal for the induction of the iNKT cell lineage transcription factor PLZF (33) and PLZF expression negatively correlates with the glycolytic potential of iNKT cells (148) potential connections between adaptor proteins and iNKT cell metabolism need to be identified.

## Author Contributions

EG prepared the figures and added comments to the manuscript. JM wrote the manuscript.

### Conflict of Interest Statement

The authors declare that the research was conducted in the absence of any commercial or financial relationships that could be construed as a potential conflict of interest.
